# Material Design of Porous Hydroxyapatite Ceramics via Inverse Analysis of an Estimation Model for Bone-Forming Ability Based on Machine Learning and Experimental Validation of Biological Hard Tissue Responses

**DOI:** 10.3390/ma17030571

**Published:** 2024-01-25

**Authors:** Shota Horikawa, Kitaru Suzuki, Kohei Motojima, Kazuaki Nakano, Masaki Nagaya, Hiroshi Nagashima, Hiromasa Kaneko, Mamoru Aizawa

**Affiliations:** 1 Department of Applied Chemistry, School of Science and Technology, Meiji University, 1-1-1 Higashimita, Tama-ku, Kawasaki 214-8571, Kanagawa, Japan; horikawash@outlook.jp (S.H.); hkaneko@meiji.ac.jp (H.K.); 2 Meiji University International Institute for Bio-Resource Research, 1-1-1 Higashimita, Tama-ku, Kawasaki 214-8571, Kanagawa, Japanhnagas@meiji.ac.jp (H.N.); 3 Department of Life Sciences, School of Agriculture, Meiji University, 1-1-1 Higashimita, Tama-ku, Kawasaki 214-8571, Kanagawa, Japan; 4 Meiji University International Institute for Materials with Life Functions, 1-1-1, Higashimita, Tama-ku, Kawasaki 214-8571, Kanagawa, Japan

**Keywords:** hydroxyapatite, bone formation rate, machine learning, inverse analysis, experimental validation

## Abstract

Hydroxyapatite and β-tricalcium phosphate have been clinically applied as artificial bone materials due to their high biocompatibility. The development of artificial bones requires the verification of safety and efficacy through animal experiments; however, from the viewpoint of animal welfare, it is necessary to reduce the number of animal experiments. In this study, we utilized machine learning to construct a model that estimates the bone-forming ability of bioceramics from material fabrication conditions, material properties, and in vivo experimental conditions. We succeeded in constructing two models: ‘Model 1′, which predicts material properties from their fabrication conditions, and ‘Model 2′, which predicts the bone-formation rate from material properties and in vivo experimental conditions. The inclusion of full width at half maximum (FWHM) in the feature of Model 2 showed an improvement in accuracy. Furthermore, the results of the feature importance showed that the FWHMs were the most important. By an inverse analysis of the two models, we proposed candidates for material fabrication conditions to achieve target values of the bone-formation rate. Under the proposed conditions, the material properties of the fabricated material were consistent with the estimated material properties. Furthermore, a comparison between bone-formation rates after 12 weeks of implantation in the porcine tibia and the estimated bone-formation rate. This result showed that the actual bone-formation rates existed within the error range of the estimated bone-formation rates, indicating that machine learning consistently predicts the results of animal experiments using material fabrication conditions. We believe that these findings will lead to the establishment of alternative animal experiments to replace animal experiments in the development of artificial bones.

## 1. Introduction

As the global population is rapidly aging, an increase in healthy life expectancy is required [1]. In order to prolong healthy life expectancy, the maintenance and recovery of locomotor functions are crucial, for which orthopedic bone regeneration is effective [2]. Bone grafting is necessary for the treatment of bone defects caused by tumors and various diseases. Autologous bone grafting is the golden standard [3]; however, numerous complications and secondary invasions occur after extraction from the donor site [4,5]. Therefore, artificial bone grafts have become more common, and artificial bones based on bioceramics have been studied.

Hydroxyapatite (Ca_10_(PO_4_)_6_(OH)_2_; HAp) is one of the bioactive ceramics and β-tricalcium phosphate (β-Ca_3_(PO_4_)_2_; β-TCP) is one of the bioresorbable ceramics, which have been clinically applied as an artificial bone material [6,7,8,9]. These porous bioceramics allow biological bone to penetrate the pores of implanted material and integrate with biological bone tissue. Controlling microstructure, such as pore size, porosity, and pore volume, can affect the bone-forming ability [10,11,12].

We have successfully synthesized and succeeded in the single-crystal apatite fiber (AF) and calcium phosphate fiber (CPF) with various Ca/P molar ratios by homogeneous precipitation using urea [13,14]. Furthermore, apatite-fiber scaffolds (hereafter, AFS) and β-tricalcium phosphate-fiber scaffolds (TFS) with macro- and microporous structures have been successfully developed by adding carbon beads (CB) as a pore-forming agent and sintering [15,16]. We have also succeeded in developing porous apatite ceramics that include bone minerals (BoneHAp) by adding six types of ions to HAp [17]. During the development of these artificial bone materials, it is necessary to verify the efficacy and safety of the materials through animal experiments. However, these processes are costly, time-consuming and require animal sacrifice [18].

The use of machine learning to predict the properties and activity (objective variable Y) of compounds and materials is common. Next, using the experimental and simulation results, a regression model (Y = *f*(X)) is constructed between Y and the explanatory variables X, which represent the experimental conditions. This model makes it possible to predict the Y values under new experimental conditions without experimentation, thereby contributing to the development of more efficient and cost-effective materials. Research is being conducted to predict the physical properties of materials such as ceramics [19], polymers [20], and metals [21], and to propose experimental conditions for materials with properties that reach the target properties, thus facilitating the development of materials. Although there have been studies using machine learning to predict biomaterial structure and properties [22,23] and predict bone formation in animal experiments with materials [24], it has been impossible to predict the in vivo responses in animal experiments from bioceramic material synthesis conditions using machine learning. Although we have previously constructed a model that predicts bone-formation rates using the results of animal experiments on developed bioceramics and proposed material fabrication conditions based on desired bone-formation rates [25], we have not performed experiments based on the proposed experimental conditions or validated the validity and practicality of the models.

In this study, we constructed a model to predict the bone-forming ability of bioceramics and designed the material fabrication conditions by inverse analysis of the constructed model, in which various candidates for the material fabrication conditions and in vivo experimental conditions were input into the model, and the material properties and bone-formation rate were predicted. Additionally, porous HAp ceramics designed by inverse analysis were fabricated. We verified whether the properties of the material and the bone-forming ability of the material implanted in the porcine tibia were consistent with the estimated values of the models.

## 2. Materials and Methods

### 2.1. Dataset

**Figure 1:**
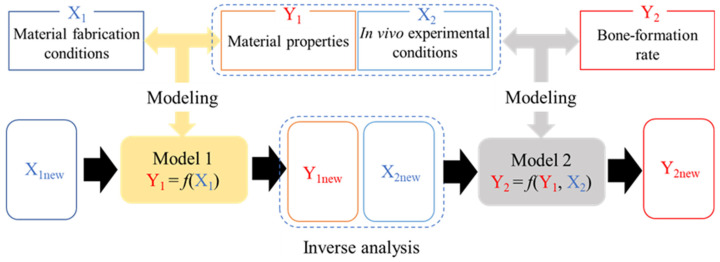
Schematic diagram of a model estimating bone-forming ability.

**Figure 2:**
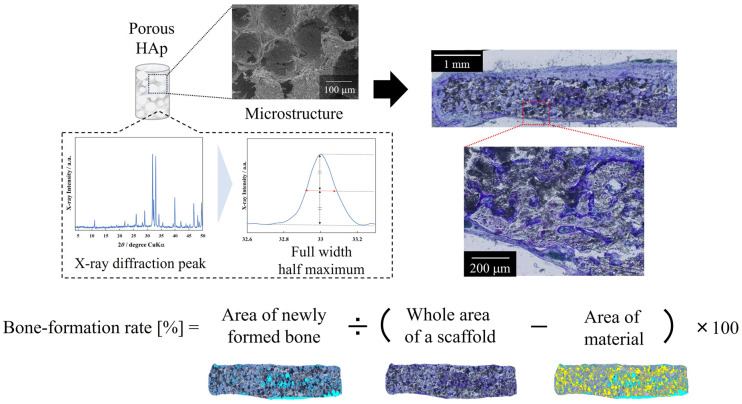
Methods for determination of bone-formation rate. Area of newly formed bone: light blue; area of material: yellow.

The dataset consists of data from material preparation to in vivo experiments on biomaterials developed in our laboratory, including AFS, TFS, and porous bone HAp ceramics. Figure 1 shows a schematic of the model that predicts material properties and bone-formation rate from material fabrication and in vivo experimental conditions. In order to predict bone-formation rate, models were constructed using material fabrication conditions, material properties, and in vivo experimental conditions. First, Model 1 (Y_1_ = *f*(X_1_)) was constructed to predict the material properties (Y_1_) from the material fabrication conditions (X_1_). Next, Model 2 (Y_2_ = *f*(Y_1_, X_2_)) was constructed to predict the bone-formation rate based on material properties (Y_1_) and in vivo experimental conditions (X_2_). The material fabrication conditions, material properties, and in vivo experimental conditions are listed in Table 1. The bone-formation rate (Y_2_) was calculated from histological images of bone tissue removed after implantation of the porcine tibia or rat calvaria using the formula in Figure 2. The data were stained with toluidine blue (TB) or villanueva bone (VB) staining. TB staining is a staining method that stains new bone blue, while VB staining is a method that differentiates the calcification stage of the bone. From stained histological images, the area of ceramics remaining after transplantation and the area of bone formation were determined, and the bone-formation rate was calculated by subtracting the area of ceramics remaining from the area of bone loss created when the specimen was transplanted.

As a preprocessing step for each variable in the dataset, autoscaling was performed to convert the mean to 0 and SD to 1.

### 2.2. Construction of Models and Verification of Predictability

Models 1 and Model 2 were constructed using Gaussian mixture regression (GMR) [25,26]. GMR is a regression analysis method with Gaussian mixture model (GMM) [27], a machine learning model that represents the relationship between *y* and the explanatory variables *x* as a superposition of multiple gaussian distributions. Also, regression and inverse analysis can be performed even for multiple *y*, and the relationship between y can be considered. When *z* connects *x* and *y*, in the GMM, the probability distribution function of the multivariate Gaussian distribution *p*(*z*) is given as follows:

(1) $$p(z)=\sum_{i=1}^{n}\pi_{i}N\;(z❘\mu_{i},\;\sum_{i}),\;\;\;\;\;\;\;\;\;\sum_{i}^{n}\pi_{i}=1$$

where *n* is the number of Gaussians, *μ_i_* and ∑*_i_* are the mean vector and variance-covariance matrix in the *i*th Gaussian. By explicitly separating *z* in Equation (1) into *x* and *y* in the GMM, the joint probability distribution of *x* and *y* can be expressed as:

(2) $$P(x,\;y)=\sum_{i=1}^{n}\pi_{i}N\;\left[x\;\;\;y\right]❘[\mu_{x,i}\;\;\;\sum_{y,i}],\left[\begin{array}{cc} \sum_{xx,i} & \sum_{yx,i}\\ \sum_{xy,i} & \sum_{yy,i} \end{array}\right]$$

where *μ_x,i_*, *μ_y,i_*, ∑*_xx,i_*, ∑*_yy,i_*, ∑*_xy,i_*, and ∑*_yx,i_*, are the mean vectors of *x* and *y*, variance–covariance matrices of *x* and *y* and the covariance matrices of *x* and *y* in the *i*th Gaussian, respectively. Estimating *y* from *x* corresponds to calculating the posterior distribution of *y* given *x* (*p*(*y|x*). *p*(*y*|*x*) can be transformed by the multiplication theorem of probability and Bayes’ theorem as follows:

(3) $$\begin{array}{c} p(y|x)=\sum_{i=1}^{n}p\;(y|x,\mu_{x,i},\;\sum_{xx,i})p{(\mu }_{x,i},\;\sum_{xx,i}\;|x)\\ =\sum_{i=1}^{n}p\;(y|x,\mu_{x,i},\;\sum_{xx,i})\frac{p{(x|\mu }_{x,i},\;\;\;\sum_{xx,i})p{(\mu }_{x,i},\;{\;\;\;\;\sum }_{xx,i})}{\sum_{j=1}^{n}p{(x|\mu }_{x,j},\;\;\;\sum_{xx,j})p{(\mu }_{x,j},\;{\;\;\;\;\sum }_{xx,j})}\\ \;\;\;=\sum_{i=1}^{n}p\;(y|x,\mu_{x,i},\;\sum_{xx,i})\frac{\pi_{i}p{(x|\mu }_{x,i},\;\;\;\sum_{xx,i})}{\sum_{j=1}^{n}\pi_{j}p{(x|\mu }_{x,j},\;\;\;\sum_{xx,j})} \end{array}$$

where *p*(*y*|*x*, *μ_x,i_,* ∑*_xx,i_*) is the multivariate Gaussian distribution of the predicted *y* values in the *i*th Gaussian. *w_x,i_*, which is the weight of the Gaussian, is given by

(4) $$w_{x,I}=\frac{\pi_{i}p{(x|\mu }_{x,i},\;\;\;\sum_{xx,i})}{\sum_{j=1}^{n}\pi_{j}p{(x|\mu }_{x,j},\;\;\;\sum_{xx,j})}$$

Substituting Equation (4) into Equation (3) gives

(5) $$p(y|x)=\sum_{i=1}^{n}w_{x,i}p\;(y|x,\mu_{x,i},\;\sum_{xx,i})$$

In *p*(*y*|*x*, *μ_x,i_,* ∑*_xx,i_*), the mean vector *m_i_*(*x*) is given by

(6) $$m_{i}(x)=\mu_{y,i}+(x-\mu_{x,i})\;\sum_{xx,i}^{-1}\sum_{xy,i}\;$$

In this study, the predicted *y* values are those with the highest weights. Additionally, since the GMM is constructed for the entire dataset, regression and inverse analysis can be performed by changing the range of *x* and *y*, considering the constraints.

To evaluate the accuracy of Model 1 and Model 2, we used double cross-validation (DCV) [25,28], a method that allows accuracy verification with a small number of samples. DCV is a nested cross-validation (CV) method used to evaluate the predictability of a model. The dataset is divided into two groups. One group is used for the validation data, and the other group is used to optimize the hyperparameters with CV and to construct a model using the obtained hyperparameters. The model is used to estimate the data for validation and to obtain DCV estimated *y* values. In this study, the dataset consists of 50 samples, with 49 samples used to build the model and the remaining one sample for predictability. This is repeated 50 times to obtain estimated *y* value for all 50 samples. The predictability was evaluated by comparing these estimated *y* values with the actual *y* values.

The coefficient of determination (*r*^2^_DCV_) and the root mean squared error (*RMSE*_DCV_) are used as evaluation indicators after DCV. *r*^2^_DCV_ and *RMSE*_DCV_ are given by:

(7) $${r^{2}}_{\mathrm{DCV}}=1-\frac{\sum_{i=1}^{n}{(y}^{\left(i\right)}-y_{DCV}^{\left(i\right)}\;{)}^{2}}{\sum_{i=1}^{n}{(y}^{\left(i\right)}-y_{mean}{)}^{2}}$$

(8) $${\mathrm{RMSE}}_{\mathrm{DCV}}=\sqrt{\sum_{i=1}^{n}\frac{{(y}^{\left(i\right)}-y_{DCV}^{\left(i\right)}{)}^{2}}{n}}$$

where *n* is the number of samples, *y*^(*i*)^ is the actual value of *i*th *y*, $y_{DCV}^{\left(i\right)}$ is the estimated value of *i*th *y*, and *y_mean_* is the average value of *y*. Higher *r*^2^_DCV_ and lower *RMSE*_DCV_ indicate higher predictive accuracy.

### 2.3. Feature Importance

We calculated the permutation feature importance (PFI), which represents how much each explanatory variable (material properties, in vivo experimental conditions) of Model 2, which predicts bone-formation rate, affected the accuracy of the model. However, PFI calculation requires training and validation data, and a small number of samples makes the calculation of feature importance unstable. Additionally, the PFI of strongly correlated features is estimated to be lower than that of other independent features. Therefore, to solve these problems and properly calculate the importance of features, we used cross-validated permutation feature importance (CVPFI) [29].

Assuming that the number of iterations is *J*, the algorithm to calculate for the CVPFI can be described as follows.

1. Calculate the correlation coefficients for all the features.
2. Calculate the absolute correlation coefficient for all the feature and set the coefficient to zero when there is no correlation.
3. During CV, for *n* = 1, 2, ⋯, *N*, where *N* is the number of CV folds, the following procedures were performed using the training and validation data (VD) at each fold.

3-1. Construct a model using the *n*th training data.

3-2. Estimate *y* for the *n*th VD, that is VD*_n_*, using the model.

3-3. For each feature *I*, which is the *i*th column of VD*_n_*, and for each repetition *j* in 1, 2, ⋯, *J*, a randomly sampled column, *I*, of the original dataset without duplication, and for each feature, *m* (the *m*th column of VD*_n_*), for which the absolute correlation coefficient between features *I* and *m*, that is, *r_i,m_*, after statistical testing is higher than 0, use a randomly sampled column, *m*, of the original data set without duplication with a probability of *r_i,m_* to generate a corrupted version of the data set CVD*_n,I,j_* and estimate the *y* values of the model.

4. Integrate the *y* values estimated during the CV for VD_1_, VD_2_, ⋯, and VD*_N_* and calculate the reference score, rscv, with the integrated *y*. This score represents the determination coefficient, *r*^2^, for a regressor.
5. Integrate the *y* estimated during the CV for CVD_1,*I,j*_, CVD_2,*I,j*_, ⋯, and CVD*_N,I,j_* and calculate the score, scv*_i,j_*, with the actualy.
6. Calculate the importance, CVPFI*_i_*, for the *i*th feature as follows:

CVPFI*_i,j_* = rscv − scv*_i,j_* (9)

(10) $${\mathrm{CVPFI}}_{i}=\frac{1}{J}\sum_{i=1}^{J}{CVPFI}_{i,j}$$

Because CVPFI can integrate the estimated *y* values and calculate *r*^2^ for a regressor using all samples, the importance can be calculated stably using CVPFI. 

Therefore, we calculate the CVPFI using the GMR used in the construction of Model 2 to interpret the precision of each explanatory variable in predicting bone-formation rates.

### 2.4. Materials Designs for New Bioceramics by Inverse Analysis of the Model

New bioceramics were designed using the constructed models. Virtual samples were generated under the following conditions: The AFS and TFS fabrication conditions (e.g., heating conditions and firing conditions) were set to known conditions. 924 samples were generated by combining 3 types of molding pressure (30–50 MPa in a increments of 10 MPa), 11 types of 150 μm CB addition ratios (0–100% in a increments of amounts of 10%), and 14 types of CB addition (50–700 mass% in a increments of amounts of 50 mass%). The virtual samples were input into Model 1 and the material properties were calculated. The calculated material properties and three in vivo experimental conditions (porcine tibia 12 weeks; rat cranial crown 2, 4 weeks) were then combined to generate 2772 samples. The virtual samples generated were input into Model 2, and the bone-formation rate was calculated. Five of the virtual samples were selected and fabricated under these conditions.

### 2.5. Fabrication of Materials

AFs were synthesized according to an earlier report [13]. The following aqueous solutions were prepared: 0.167 mol·dm^−3^ Ca(NO_3_)_2_·4H_2_O (FUJIFILM Wako Pure Chemical Co., Osaka, Japan), 0.100 mol·dm^−3^ (NH_4_)_2_HPO_4_ (FUJIFILM Wako Pure Chemical Co., Osaka, Japan), 0.500 mol·dm^−3^ (NH_2_)_2_CO (FUJIFILM Wako Pure Chemical Co., Osaka, Japan), and 0.100 mol·dm^−3^ HNO_3_ (FUJIFILM Wako Pure Chemical Co., Osaka, Japan). The Ca/P ratio was adjusted to 1.67. The AFs were mixed with a spherical CB of ~150 μm and ~20 μm diameter in a mixed solvent (ethanol/water = 1:1 [*v*/*v*]). Based on the results of the inverse analysis, the amount of CB (Nikabeads; Nihon Carbon Company, Tokyo, Japan) added and the ratio of CB with different particle sizes were determined, and uniaxial pressure molding was performed at 30 MPa. AFS were prepared by baking the compacts in a steam atmosphere at 1300 °C, with a temperature increase rate of 5 °C·min^−1^, for 5 h. AFSs with dimensions of 3.8 mm (diameter) and 7 mm (height) were used in this study. The samples were abbreviated as AFS X(Y-Z), where X is the amount of CB added (mass%), Y is the ratio of 150 μm CB added (%), and Z is the ratio of 20 μm CB added (%). Five types of AFS were fabricated.

### 2.6. Material Characterization and Validation

The AFSs were characterized as follows. Phase composition was determined by X-ray diffractometry (XRD; MiniFlex, Rigaku Co., Tokyo, Japan). The X-ray generator with CuKα was operated at 30 kV and 15 mA. Data were collected in the range of 2*θ* 4.0–50.0° with a step size of 0.04° and a count speed of 4°·min^−1^. We achieved phase identification by comparing the diffraction patterns with the International Centre for Diffraction Data standard. Fourier transform infrared (FT-IR; IR Prestige21, Shimadzu Co., Kyoto, Japan) spectra were measured using the KBr tablet method. Spectra were obtained at a 4 cm^−1^ resolution over the range of 400–4000 cm^−1^ with a scan speed of 4 mm·s^−1^. The microstructures were observed by scanning electron microscopy (SEM; JSM6390LA, JEOL Ltd., Tokyo, Japan). Compressive strength was measured using a universal test machine (Autograph AGS-J, Shimadzu Co., Kyoto, Japan) at a crosshead speed of 0.5 mm·min^−1^. Porosity was determined as follows. Firstly, the bulk density was determined from the dimensions and mass of the fabricated ceramics specimens. This value was divided by the theoretical density (for intense, 3.16 g/cm^3^ in the case of HAp) and multiplied by 100 to calculate the relative density (%). Porosity (%) was determined by subtracting the relative density (%) from 100%. The actual porosity and compressive strength were compared with the estimated values of the model.

### 2.7. In Vivo Evaluation of Materials Using a Pig Tibia Model and Validation

We used a miniature pig weighing 83.8 kg to evaluate the bone-forming ability of AFSs in vivo. Subsequently, the right tibia of the pig was exposed and a 3.8 mm diameter cylindrical defect was opened in the tibial epiphysis. A dry, heat-sterilized AFS was placed into the defect. The pig had access to a standard laboratory diet and water throughout the study. Surgical procedures were performed according to the Animal Care and Use Committee of Meiji University (approval number: MUIACUC 2020-11).

After implantation for 12 weeks, the pig was sacrificed, and the tibiae were removed. Noncalcified polished sections were prepared and observed under an optical microscope after VB staining. Bone-formation rates were measured using image analysis software (WinROOF ver 6.4.0, Mitani Co., Fukui, Japan). Subsequently, we verified whether the measured bone-formation rates were in agreement with the estimated values of the model.

## 3. Results and Discussion

### 3.1. Construction of Model 1

The Porosity, compressive strength, and full width at half maximum (FWHM) of the characteristic X-ray diffraction maximums, were predicted from the material fabrication conditions. Using the peak of the International Centre for Diffraction Data (ICDD) card, FWHM was calculated from the XRD data as the (2 1 1) and (3 0 0) peaks for HAp (#09-432) and the (2 2 0) and (0 2 10) peaks for β-TCP (#09-169). The highest peak in the XRD of HAp is (2 1 1), which is a shared plane with (1 2 1). In our present experiment, the (2 1 1) peak was detected as a single diffraction line. Therefore, we used this peak to obtain the FWHM, and then used it as one of the features X in an estimation model for bone-forming ability.

**Figure 3:**
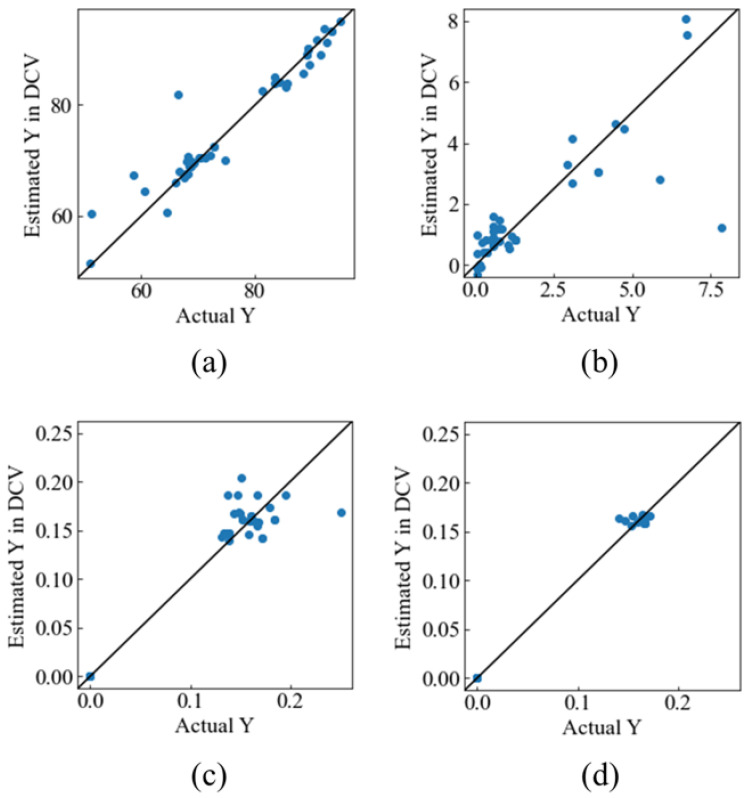
Actual Y_1_ versus estimated Y_1_ in DCV: (**a**) Porosity, (**b**) compressive strength, (**c**) FWHM_211_, (**d**) FWHM_220_.

The plots of the actual material property versus the predicted material property in DCV are shown in Figure 3. The calculated values of the *r*^2^_DCV_ and *RMSE*_DCV_ are shown in Table 2. DCV results show that approximately 90% of porosity, 70% of the compressive strength, and 90–99% of FWHM were predicted for the new data. Outliers were identified in the scatter plots for compressive strength. Furthermore, in compressive strength tests, there tends to be a large variation in the measured values even under the same experimental conditions. In this study, the model was constructed using average values without considering the variation. This suggests that some samples were off the diagonal due to variations caused by individual differences in materials. These results show that GMR can predict multiple material properties simultaneously with high predictability.

### 3.2. Construction of Model 2

**Figure 4:**
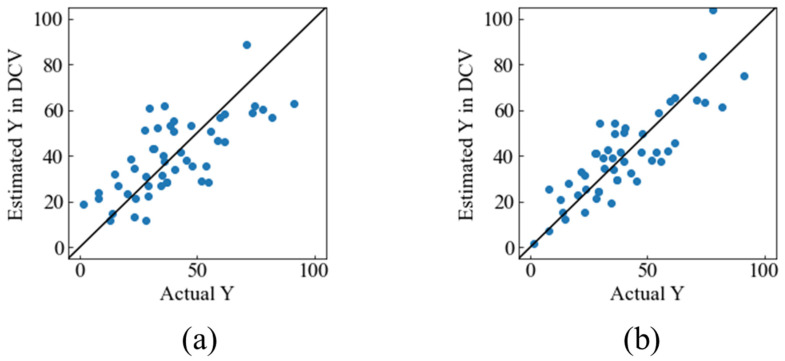
Actual Y_2_ versus estimated Y_2_ in DCV: (**a**) Without FWHM, (**b**) with FWHM.

The bone-formation rates were predicted on material properties and in vivo experimental conditions. We compared the predictability of Model 2 with and without FWHM. The plots of the actual bone-formation rate versus the predicted bone-formation rate in DCV are shown in Figure 4. The calculated values of the *r*^2^_DCV_ and *RMSE*_DCV_ are also shown in Table 3. Without FWHM, the predictability was approximately 50%. With the addition of the FWHM, we found that the model could predict approximately 70% of the new data. This result suggests that FWHM is an important factor in predicting bone-formation rates. When FWHM is an explanatory variable, information on the crystal structure of the material can be included in the model. Since the crystalline structure of the material affects its bone-forming ability, the addition of FWHM may have improved accuracy. As a result, Model 2 was to predict complex in vivo reactions with 70% predictability.

### 3.3. Feature Importance

**Figure 5:**
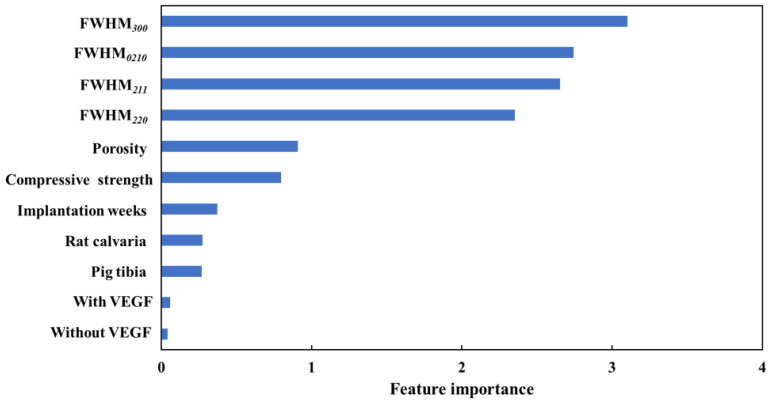
Feature importance of Model 2.

In order to determine how much each feature of Model 2, which predicts bone-formation rate, affects the accuracy, the feature importance was calculated. The feature importance was calculated as the CVPFI using GMR. Model 2 was constructed using material properties, in vivo experimental conditions, vascular endothelial growth factor (VEGF), and FWHM 4 peaks for bone formation rate, and CVPFI values were calculated. The calculated feature importance is shown in Figure 5. The FWHM 4 peak was found to be the most important. The four FWHMs were equally important. FWHM’s high importance suggests that the identification of the crystalline phase and the slight differences in crystallinity affect the model. Next, using the half width, the crystallite size can be calculated using Scherrer’s equation [30], where a large crystallite size indicates a small FWHM. However, when the FWHM is small, Scherrer’s equation cannot be applied to determine the crystalline size due to an increase in the error. The FWHM of the material included in these data is also small and Scherrer’s equation could not be applied. Therefore, by using half-width instead of crystallite size, the crystallinity of the material could be expressed as the explanatory variable X. Different crystallite size values along crystallographic planes (2 1 1) and (3 0 0) for HAp or (0 2 10) and (2 2 0) for β-TCP may indicate on anisotropic crystallite shape. Furthermore, the equal importance of the four FWHMs suggests that the relationship between these two HAp and β-TCP peaks contributed to the model. It is also suggested that the model could include the crystalline morphology of the material. When the importance of the material properties (porosity and compressive strength) and the in vivo experimental conditions were compared, the material properties were found to be more important. This shows that the material properties contribute significantly to the model. In other words, material properties have a greater influence on bone-forming ability than in vivo experimental conditions (the week of implantation and implantation animal). Porosity is a property that describes the microstructure and has been reported in the past to affect bone-forming ability, which is consistent with its importance [11,15,16]. Compressive strength is a property that depends on porosity. It can be inferred that the importance of compressive strength increases with the importance of porosity.

### 3.4. Materials Designs for New Bioceramics by Inverse Analysis of Model

A total of 2772 virtual samples were generated under various constraints. Material properties were calculated by inputting the generated samples into Model 1. Here, FWHM was not used, and the crystalline phase was added to the variables as a dummy variable (0: HAp, 1: β-TCP) to classify the crystalline phase. Specifically, AFS was set to 0 (HAp) and TFS to 1 (β-TCP) and calculated as the crystalline phase, and the calculated material properties and in vivo experimental conditions were input into Model 2 to calculate bone-formation rate. The applicability domain (AD) [31] was set to investigate the reliability of the virtual sample. The distance between the existing and designed material fabrication conditions was calculated using the *k*-nearest neighbor (*k*-NN) method [32], a method of averaging the distance to new data. *k*-NN calculations used Euclidean distance, and *k* was set to 1 due to the small number of training samples. The distance between the 2772 generated samples and the closest known distance was calculated, and the 99.7% value was used as the threshold for AD. Of the 2772 samples generated, 2733 were identified as samples within AD. Five AFSs were selected among the samples within AD. The experimental conditions, calculated material properties, and calculated bone-formation rates for the selected samples are presented in Table 4.

### 3.5. Experimental Validation of Material Properties

In this section, we describe the results of AFSs prepared of the experimental conditions presented in the inverse analysis and compare them with the properties of their actual and predicted materials. Material XRD patterns showed that all AFSs were single-phase HAp. The FT-IR spectra of AFS showed absorptions of PO_4_^3−^ groups at 1300–900, 600, and 570 cm^−1^ and absorptions of OH^−^ groups at 3570 cm^−1^. SEM images of AFSs that contain many macropores because of the burning of CBs and many interconnected micropores derived from entangled AFs.

**Figure 6:**
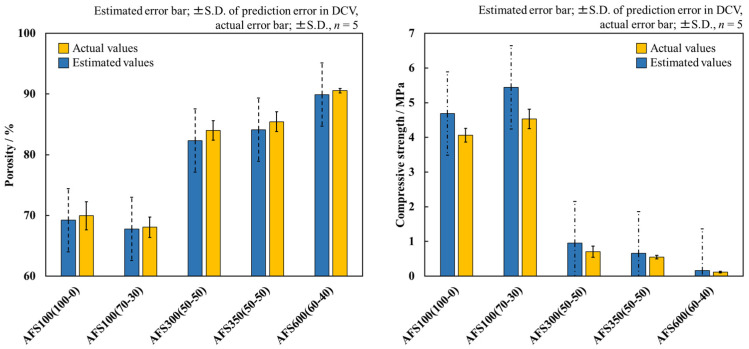
Comparison of actual porosity and compressive strength with estimated ones. Error bars for actual values represent the standard deviation (*n* = 5); error bars for estimated values represent the standard deviation of error between estimated values and actual ones in DCV.

Figure 6 left shows the comparison of the actual and estimated porosities of AFS. The error bar of the estimated porosity is the SD of the error between the actual and predicted porosities for each sample in DCV (SD; ±3.09). If the actual value exists within the error range of the estimated value, the actual value is defined as consistent with the estimated value. The porosity increased with increasing amount of CB added. The porosity also increased with the addition of 150 μm CB. This trend was similar to that of the estimated porosities of the model. The actual porosities of all AFSs were within the error range of the estimated porosities and the values were generally in agreement. In the DCV, the predictability for approximately 90% of the unknown data suggests that the actual and estimated values were mostly in agreement.

Figure 6 right shows the comparison of the actual compressive strength and the estimated compressive strength of the AFSs. The error bar of the estimated compressive strength is the SD of the error between the actual and the estimated compressive strengths for each sample in DCV (SD; ±1.32). The compressive strength decreased as the amount of added CB increased. Additionally, the compressive strength also increased with the ratio of CB with a smaller particle size. This trend was the same as the estimated compressive strength of the model, and the actual compressive strength of all AFSs was within the error range of the estimated compressive strength. However, for the two AFSs with a high compressive strength, there was a large error between the actual and estimated values. The actual values of the material with a high compressive strength exhibited significant variation due to individual differences in the materials and measurement errors. Additionally, assuming the same trend in the dataset, individual differences in materials and measurement errors could be the cause of the prediction errors of Model 1.

A comparison of porosity and compressive strength showed relatively good agreement between the actual and estimated values. The dataset used in this study contains a large amount of data on porous bioceramics with different conditions for the addition of pore-forming agents. In the inverse analysis, virtual samples were generated by varying the pore-forming agent addition conditions and the molding pressure. This indicates that the virtual samples were generated close to the samples included in the dataset. In fact, 98% of the virtual samples generated were within AD. It restricted inverse analysis based on the dataset results in good agreement between the estimated and actual values. These results suggest that porous ceramics with the desired strength and porosity can be designed and fabricated.

### 3.6. In Vivo Evaluation of Materials and Validation

**Figure 7:**
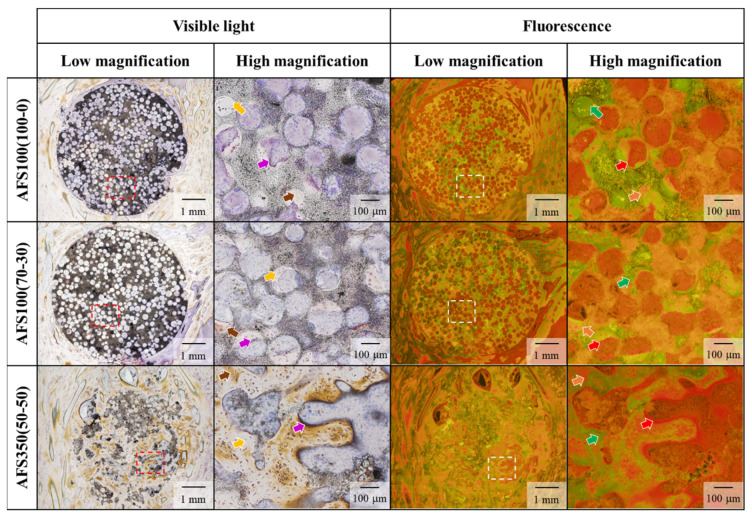
VB staining of AFSs. High magnification: highly magnified images of dashed square area. Visible light: osteoid purplish red; hypocalcified bone: brown; calcified bone: light orange. Fluorescence: osteoid: red; hypocalcified bone: orange; calcified bone: green.

Among the five AFSs fabricated, three kinds of AFSs were used in animal experiments: (1) AFS100(100-0), (2) AFS100(70-30), and (3) AFS350(50-50). Histological images of VB staining of pig tibia in visible light and fluorescence are shown in Figure 7. It was observed that all AFSs were directly bound to the newly formed bone of the host. Additionally, good bone formation was observed around and inside the material, which confirmed its excellent osteogenic potential. Fluorescence imagery also showed that two AFSs with a porosity of approximately 70% formed a large amount of calcified bone.

**Figure 8:**
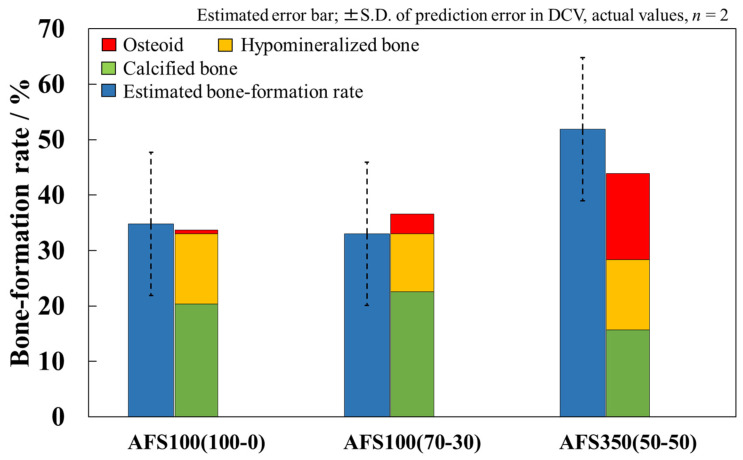
Comparison of actual bone-formation rate with estimated ones. Error bars for estimated values represent the standard deviation of error between estimated bone-formation rates and actual ones in DCV.

Figure 8 shows a comparison of the bone-formation rates calculated by image analysis with estimated bone-formation rates. The error bar of the estimated bone-formation rate is the SD of the error between the actual and the estimated bone-formation rate for each sample in DCV (SD; ±12.93). The AFS350(50-50) bone-formation rate was the highest among the porous AFS specimens. The trend between actual and estimated bone-formation rates was mostly consistent. In particular, the actual bone-formation rate of AFS100(100-0) was almost in agreement with the estimated bone-formation rate. Actual bone-formation rates in all AFSs were within the error range of estimated bone-formation rates. This error could be attributed to errors in predictability and individual differences among experimental animals, i.e., the results showed that the predictions were correct within the error range due to individual animal differences.

In contrast, there were no differences in the estimated bone-formation rate between AFS100(100-0) and AFS100(70-30). Only porosity was included as an explanatory variable for the microstructure. It has been reported that not only porosity, but also pore diameter and pore volume affect bone-forming ability [10,12,33], and it is possible that porosity alone does not explain the bone-formation rate. In the future, we aim to further improve the predictability by including more detailed data on the microstructure, such as pore size distribution, and by extracting features through image analysis using SEM images and adding them as explanatory variables.

In summary, the use of consistent data from material synthesis to animal experiment results in the model construction not only enabled prediction by machine learning, but also enabled agreement in experimental validation. We believe that further improvement of the accuracy of the model can be applied to other artificial bones (e.g., commercially available artificial bones) in the future.

## 4. Conclusions

We used machine learning to construct Model 1 (Y_1_ = *f*(X_1_)) to predict material properties (Y_1_) from material fabrication conditions (X_1_) and Model 2 (Y_2_ = *f*(Y_1_, X_2_)) to predict bone-formation rate (Y_2_) from material properties (Y_1_) and in vivo experimental conditions (X_2_). The predictability of Model 2 was improved by adding FWHM to the explanatory variables. Based on the feature importance, the FWHM was the most important in predicting the bone-formation rate, as far as we were able to determine in this work. This suggests that the crystalline phase and crystallinity affect the bone-forming ability. Through an inverse analysis of the constructed models, we proposed the fabrication conditions for new porous hydroxyapatite ceramics and then fabricated the materials under the conditions. The actual material properties were relatively close to the estimated material properties. The created materials were implanted into the porcine tibia and the bone-formation rate was calculated. The actual bone-formation rates existed within the error range of the estimated bone-formation rates. We believe that this research will enable us to propose artificial bone materials that match the individual patients in some experiments. Furthermore, we propose a novel process of artificial bone material development that consistently predicts the in vivo response of material fabrication to animal testing with the help of machine learning, leading to the establishment of an alternative method of animal testing to replace animal testing.

## Figures and Tables

**Table 1:** X_1_, Y_1_, X_2_, Y_2_ used in construction of models.

Variables	Variable Names	Variables	Variable Names
X_1_	Ca(OH)_2_/mol·dm^−3^	X_1_	20 μm CB rate/%
	MgCl_2_·6H_2_O/mol·dm^−3^		Molding pressure/MPa
	NaCl/mol·dm^−3^		Firing tempreture/°C
	KCl/mol·dm^−3^		Firing atmosphere
	(NH_4_)_2_CO_3_/mol·dm^−3^		(0: air, 1: steam, 2: steam in carbonate gas)
	H_3_PO_4_/mol·dm^−3^	Y_1_	Porosity/%
	NH_4_F/mol·dm^−3^		Compressive strength/MPa
	Ball mill grinting time/h		Half-width of 4 peaks/degree
	(NH_4_)_2_HPO_4_/mol·dm^−3^	X_2_	Implantation periods/weeks
	Si(OC_2_H_5_)_4_/mol·dm^−3^		Implantation animals
	Heating tempreture/°C		(0: pig, 1: rat)
	Heating time/h		Vascular endothelial growth factor adding
	Amount of carbon beads (CB)/mass%		(0: without, 1: with)
	150 μm CB ratio/%	Y_2_	Bone-formation rate/%

**Table 2:** *r*^2^_DCV_ and *RMSE*_DCV_ for Each Feature Model 1.

Material Properties	*r* ^2^_DCV_	*RMSE*_DCV_
Porosity	0.933	3.02
Compressive strength	0.745	0.12
FWHM_211_	0.935	0.02
FWHM_220_	0.996	0.01

**Table 3:** *r*^2^_DCV_ and *RMSE*_DCV_ for without or with FWHM Model 2.

Feature	*r* ^2^_DCV_	*RMSE*_DCV_
Without FWHM	0.497	14.33
With FWHM	0.689	11.30

**Table 4:** Results of inverse analysis.

X_1_		Y_1_			X_2_		Y_2_
Amount of CB [mass%]	150 μm CB Rate [%]	Porosity [%]	Compressive Strength [MPa]	Crystalline Phase	Implantation Weeks	Implantation Animal	Bone-Formation Rate [%]
100	100	70.0	4.7	HAp	12	Pig	37.0
100	70	68.1	5.4				36.8
300	50	84.0	0.9				49.6
350	50	85.4	0.7				51.6
600	60	90.5	0.2				59.4

## Data Availability

The data presented in this study are available on request from the corresponding author. The data are not publicly available due to privacy and ethical constraints.
